# Efficacy of Qishen Yiqi Drop Pill for Chronic Heart Failure: An Updated Meta-Analysis of 85 Studies

**DOI:** 10.1155/2020/8138764

**Published:** 2020-09-22

**Authors:** Hao Wang, Lixia Li, Xiaochun Qing, Shouyan Zhang, Shulong Li

**Affiliations:** Department of Cardiology, Luoyang Central Hospital Affiliated to Zhengzhou University, Luoyang, Henan Province, China

## Abstract

**Background:**

Despite evidence for beneficial effects of Qishen Yiqi Drop Pill (QSYQ) on congestive heart failure, the majority of studies are based on insufficient sample sizes. The aim of this study was to evaluate the therapeutic effects of QSYQ using a meta-analysis approach. *Methodology/Principal Findings*. All relevant studies published before December 31, 2019, were identified by searches of various databases with key search terms. In total, 85 studies involving 8,579 participants were included. The addition of QSYQ to routine Western medicine increased 6-minute walking distance (SMD = 2.08, 95% CI: 1.72–2.44, *p* < 0.001), left ventricular ejection fraction (SMD = 1.05, 95% CI: 0.87–1.23, *p* < 0.001), and cardiac index (SMD = 1.44, 95% CI: 0.92–1.95, *p* < 0.001) and reduced brain natriuretic peptide (SMD = −2.28, 95% CI: -2.81 to -1.76, *p* < 0.001), N-terminal prohormone of brain natriuretic peptide (SMD = −2.49, 95% CI: -3.24 to -1.73, *p* < 0.001), left ventricular end-diastolic dimensions (SMD = −0.92, 95% CI: -1.25 to -0.59, *p* < 0.001), and left ventricular end-systolic dimensions (SMD = −0.55, 95% CI: -0.89 to -0.21, *p* < 0.001). The results were stable in subgroup analyses and sensitivity analyses.

**Conclusions:**

Our current meta-analysis indicated that QSYQ combined with Western therapy might be effective in CHF patients. Further researches are needed to identify which subgroups of CHF patients will benefit most and what kind of combination medicines work best.

## 1. Introduction

Most cardiovascular diseases eventually progress to chronic heart failure (CHF) [1]. Based on Framingham data, the lifetime risk of developing CHF is 20%, and the incidence increases with age, with a steep rise from 1.4–1.9% among middle-aged individuals to 12.8–14.7% among octogenarians [2]. As the population is aging, CHF is becoming the leading public health challenge worldwide. About half of individuals who are diagnosed with heart failure die within five years [3]. However, before death, CHF patients have to encounter constantly worsening and increasingly frequent suffering of symptoms caused by CHF, such as dyspnea, fatigue, edema, and a reduced ability to exercise [4, 5]. In addition to a reduced quality of life, CHF also results in heavy economic burden to both families and society [6, 7].

Qishen Yiqi Drop Pill (QSYQ) is a traditional Chinese medicine. It is composed of extracts of four herbaceous plants, *Salvia miltiorrhiza* Bunge (“danshen” in Chinese), *Panax notoginseng* (“Sanqi” in Chinese), *Astragalus membranaceus* (Fisch.) Bunge (“huangqi” in Chinese), and *Dalbergia odorifera* T. Chen (“Jiangxiang” in Chinese) [8]. It is an effective therapeutic agent for coronary artery disease [9]. Recently, extensive studies have explored the effects of QSYQ on CHF [10–12]. However, the results of these studies are not sufficient to establish standards for evidence-based practice, as most are limited by small sample sizes and differences in treatment duration. To the best of our knowledge, only one meta-analysis has evaluated the effects of QSYQ on CHF, including only 12 studies with a Jadad score of ≥2 [13]. Quality assessment is essential for meta-analyses; however, the Jadad scale is not suitable for study exclusion, as it contains no mention of allocation concealment, which is important in the evaluation of an RCT's internal validity [14]. Furthermore, many studies have been published since August 2018, the cutoff for the previous meta-analysis. Accordingly, in this study, we conducted an updated meta-analysis to explore the effectiveness of QSYQ in patients with CHF.

## 2. Methods

### 2.1. Data Sources and Study Identification

All studies exploring the effects of QSYQ in patients with CHF published before December 31, 2019, were included. Studies were identified by searching the PubMed, Cochrane Library, Wanfang Database, China Scientific Journal Database (VIP), China National Knowledge Infrastructure (CNKI), and China Biology Medicine (CBM) databases using different combinations of terms, including “QiShenYiQi”, “Qishen Yiqi”, “QSYQ”, “Qishen Yiqi Drop Pill”, “heart failure”, and “cardiac dysfunction”. All studies retrieved in this search were reviewed by two experienced researchers (HW and LL) independently and in parallel to minimize subjective selection bias. Divergences were adjudicated by discussion with a third investigator (XQ). Studies were excluded if they fulfilled the following criteria: (i) animal experiments and mechanistic studies; (ii) clinical studies but using non-RCT design; (iii) the study population was not patients with CHF; and (iv) data were repetitive or unavailable. There was no limitation with respect to language and region.

### 2.2. Data Extraction

Data were double entered by two investigators (HW and LL) independently. For every study, the following information was extracted: name of the first author, year of publication, paper title, journal name, enrolled start and end date, region of the study population, sample size of QSYQ groups and controls, treatment course, therapy in the control group, published language, methodological information (for quality assessment), and cardiac function-related parameters (6-minute walking distance (6MWD), brain natriuretic peptide (BNP), N-terminal prohormone of BNP (NT-pro BNP), left ventricular ejection fraction (LVEF), left ventricular end-diastolic dimensions (LVEDD), left ventricular end-systolic dimensions (LVESD), and cardiac index). If concrete data could not be obtained, the corresponding authors were contacted by e-mail or other methods.

### 2.3. Quality Assessment

A modified Jadad scale was used to evaluate the quality of included studies, which referred to four aspects: randomization, concealment of allocation, double blinding, and withdrawals and dropouts. The scores were 2 points, 2 points, 2 points, and 1 point, respectively. The quality of the RCT receiving 1-3 points was evaluated as low, while 4-7 points was high [15, 16].

### 2.4. Ethics Statement

Ethical approval is not applicable for the meta-analysis as it is a secondary study.

### 2.5. Data Analyses

The meta-analysis was conducted using the meta package (Schwarzer, 2007; Balduzzi et al. 2019) for R software version 4.0.2. The outcomes included 6MWD, BNP level, NT-pro BNP level, LVEDD, LVESD, LVEF, and cardiac index. The standardized mean difference (SMD) was used to enable comparisons because means differed widely among studies owing to the substantial variation in cardiac function among participants. An SMD value of 0.2, 0.5, and 0.8 presents small, medium, and large effect sizes, respectively [17]. Before combination, heterogeneity was evaluated based on the *I*^2^ metric of inconsistency and the *χ*^2^-based Cochran *Q* test. The value of *I*^2^ reflected the proportion of the impacts caused by between-study heterogeneity rather than sampling error [18]. In the absence of notable heterogeneity (*I*^2^ < 50%), a fixed effect model was used to calculate the effect size; when heterogeneity was detected (*I*^2^ ≥ 50%), a random effects model was used. Detailed differences between the two models were described in our previous studies [19]. The *z*-test was used to assess the combined statistical outcomes. As the value of 95% CI of effective size only reflects the average level of the current included studies, in order to expect the true effect of QSYQ used in future studies, 95% prediction interval was also calculated according to the formula introduced by IntHout [20].

The treatment dose was the same in all studies; however, the treatment duration differed. Therefore, a subgroup analysis was conducted by treatment course. Durations of 1–4 weeks, 5–8 weeks, and ≥9 weeks were defined as short, intermediate, and long treatment courses, respectively. To confirm the results of this analysis, a meta-analysis of studies classified as high quality (Jadad score ≥ 4 points) was further performed. As there is still high heterogeneity among studies in subgroups stratified by treatment course and study quality, a Galbraith plot was used to identify potential sources of heterogeneity, and data were reevaluated after excluding outlier studies [21]. Publication bias was evaluated for parameters reported in at least 10 studies based on funnel plots, Begg's rank correlation test, and Egger's linear regression test implemented in Stata version 12.0 (Stata, College Station, TX, USA). If publication bias was identified, the trim-and-fill method was used for correction by conservatively imputing hypothetical negative unpublished studies to mirror the positive studies that cause funnel plot asymmetry [22]. A two-tailed value of *p* < 0.05 was considered statistically significant.

## 3. Results

### 3.1. Characteristics of the Included Studies

**Figure 1:**
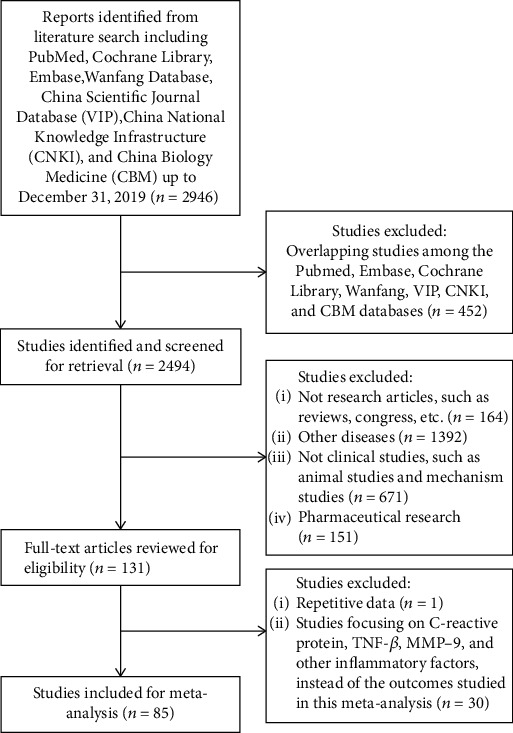
Flow diagram of studies included in this meta-analysis.

A total of 2,946 potential studies were retrieved by the initial search. Among these studies, 2,815 were discarded by screening titles, abstracts, or full-length texts, as summarized in Figure 1. Ultimately, 85 studies with a total of 8,579 participants (4,333 participants treated with a combination of QSYQ and routine Western medicine; 4,246 participants treated with only routine Western medicine as a control group) were included in the final analysis. The routine Western medicine mainly contained angiotensin-converting enzyme inhibitor (ACEI), loop diuretic (LD), aldosterone receptor antagonists (MRAs), digitalis, and *β*-adrenergic blocker (BB).

The characteristics of the 85 studies are summarized in Table S1 (supplementary data). In brief, all of the studies were published in Chinese. Twenty-five studies were defined as high quality by the modified Jadad scale (score ≥ 4). Both men and women were enrolled in all studies. Ischemic heart disease was the main cause of heart failure, and other causes included valvular heart disease, dilated cardiomyopathy, and hypertensive myocardiopathy, as well as pulmonary heart disease. The majority of patients were in NYHA functional classes II to IV, and half were NYHA III to IV. Patients with CHF in the experimental groups were all treated with QSYQ (0.5 g once orally, 3 times daily) combined with routine Western drugs, and those in the control group were treated with routine Western drugs only. In total, 29, 24, and 32 studies reported short, intermediate, and long treatment durations, respectively.

### 3.2. Effects of QSYQ on Observed Outcomes

**Figure 2:**
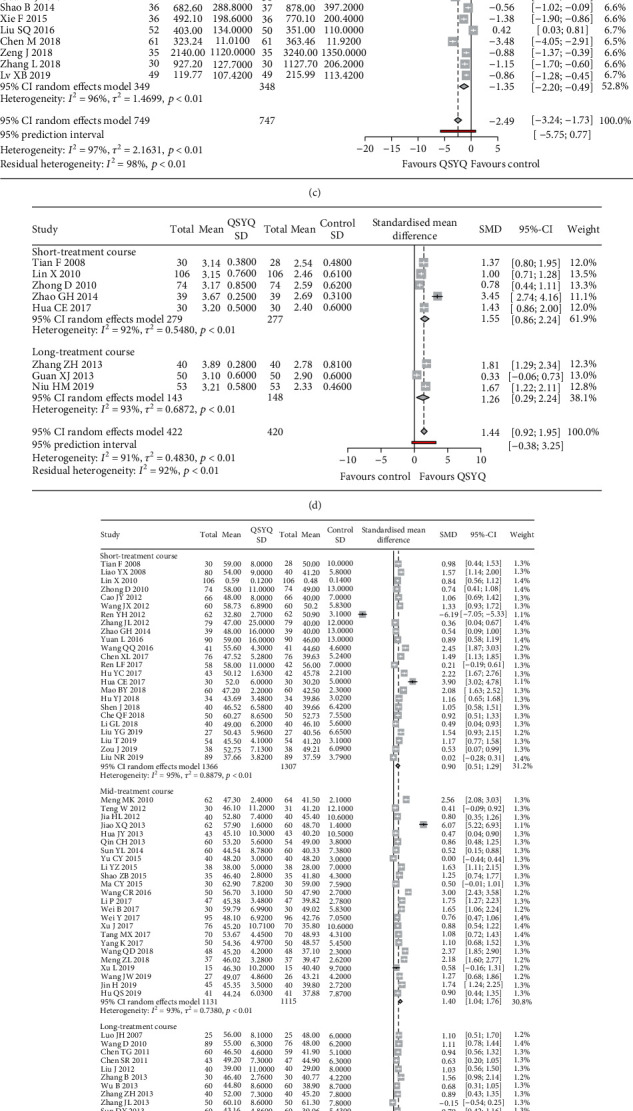
Forest plot of the meta-analysis of QSYQ addition on outcome parameters: (a) the forest plot for 6MWD; (b) for BNP; (c) for NT-pro BNP; (d) for cardiac index; (e) for LVEF; (f) for LVEDD; (g) for LVESD.

As shown in Figures 2(a)–2(g), the addition of QSYQ to conventional Western therapies in patients with CHF significantly increased 6MWD (SMD = 2.08, 95% CI: 1.72–2.44, *p* < 0.001); improved left ventricular enlargement, as evidenced by decreased LVEDD and LVESD (LVEDD: SMD = −0.92, 95% CI: -1.25 to -0.59, *p* < 0.001; LVESD: (SMD = −0.55, 95% CI: -0.89 to -0.21, *p* < 0.001); increased LVEF (SMD = 1.05, 95% CI: 0.87–1.23, *p* < 0.001); elevated cardiac index (SMD = 1.44, 95% CI: 0.92–1.95, *p* < 0.001); and decreased levels of BNP and NT-pro BNP (BNP: SMD = −2.28, 95% CI: -2.81 to -1.76, *p* < 0.001; NT-pro BNP: SMD = −2.49, 95% CI: -3.24 to -1.73, *p* < 0.001). In addition, we conducted a subgroup analysis stratified by treatment duration. Consistent results were observed among all subgroups for the parameters 6MWD, LVEF, BNP, NT-pro BNP, and cardiac index. In a sensitivity analysis of high-quality studies (JADAD score ≥ 4 (on a 7-point scale)) in both overall and subgroups, QSYQ administration in addition to conventional Western therapy significantly reduced levels of BNP and NT-pro BNP and improved cardiac function as well as exercise tolerance (Table 1).

In order to make further exploration of the true effect of QSYQ in the future practice settings, 95% prediction interval was also calculated. As shown in Figure 2(a), the effect size of 6MWD is 2.08 (95% CI 1.72-2.44), but its 95% prediction interval is -0.22 to 4.38. The prediction interval contains zero and values below zero. It indicated that QSYQ may not always be beneficial in clinical application. It might be even slightly harmful in some cases. The similar phenomena were also observed in the value of 95% prediction interval of BNP (-5.56 to 1.00), NT-pro BNP (-5.75 to 0.77), cardiac index (-0.38 to 3.25), LVEF (-0.52 to 2.62), and LVEDD (-2.97 to 1.12), as well as LVESD (-2.39 to 1.28).

### 3.3. Heterogeneity Analysis

**Figure 3:**
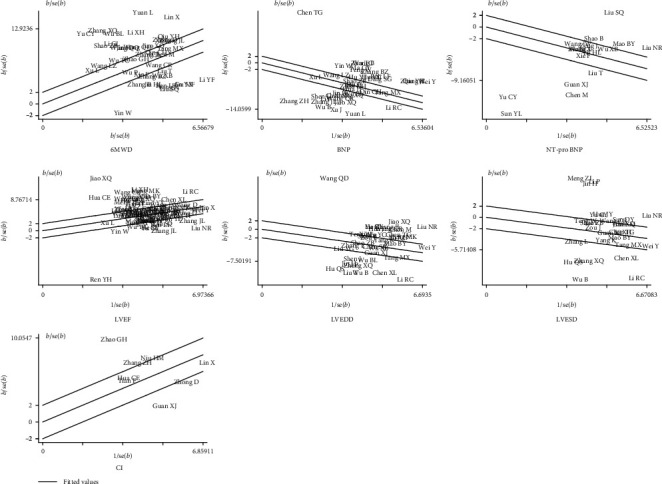
Galbraith plots for all the parameters.

Significant heterogeneity was observed in all analyses, including analyses of subgroups stratified by treatment course and study quality. Accordingly, a Galbraith plot was conducted. We identified 31 studies, 27 studies, 9 studies, 36 studies, 26 studies, 15 studies, and 5 studies, respectively, as the main sources of heterogeneity for 6MWD, BNP, NT-pro BNP, LVEF, LVEDD, LVESD, and cardiac index (Figure 3). The heterogeneity was effectively removed or decreased after the exclusion of these outlier studies, but the SMD values and 95% CIs did not change substantially (6WMD: SMD = 1.71, 95% CI = 1.54, 1.87, *p*_SMD_ < 0.001, *p*_heterogeneity_ = 0.15; BNP: SMD = −1.93, 95% CI = −2.12, −1.75, *p*_SMD_ < 0.001, *p*_heterogeneity_ = 0.28; NT-pro BNP: SMD = −1.06, 95% CI = −1.25, −0.87, *p*_SMD_ < 0.001, *p*_heterogeneity_ = 0.76; LVEF: SMD = 0.93, 95% CI = 0.87, 1.00, *p*_SMD_ < 0.001, *p*_heterogeneity_ = 0.10; LVEDD: SMD = −0.73, 95% CI = −0.86, −0.61, *p*_SMD_ < 0.001, *p*_heterogeneity_ = 0.27; LVESD: SMD = −0.54, 95% CI = −0.66, −0.43, *p*_SMD_ < 0.001, *p*_heterogeneity_ = 0.20; and cardiac index: SMD = 1.17, 95% CI = 0.88, 1.46, *p*_SMD_ < 0.001, *p*_heterogeneity_ = 0.28).

### 3.4. Publication Bias

**Figure 4:**
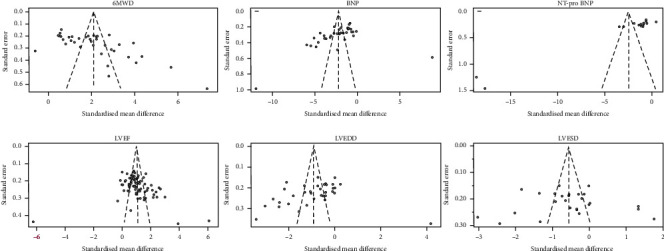
Funnel plots for all the parameters.

**Figure 5:**
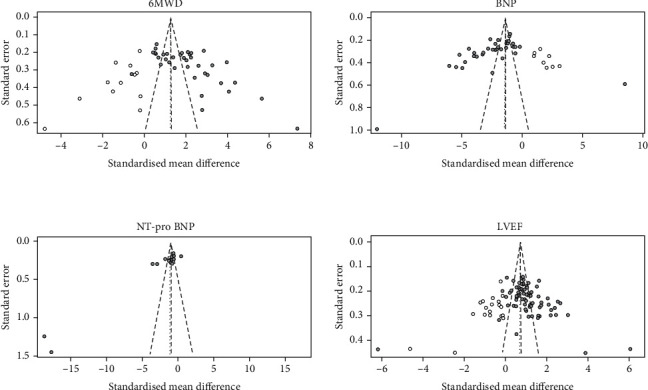
Filled funnel plots for all the parameters.

A visual inspection of funnel plots for 6MWD, BNP, NT-pro BNP, and LVEF revealed asymmetry (Figure 4). Both Begg's test and Egger's test provided evidence for publication bias (6MWD: Begg's test *z* = 3.73, *p* < 0.001, and Egger's test *p* < 0.001; BNP: Begg's test *z* = 4.02, *p* < 0.001, and Egger's test *p* = 0.010; NT-pro BNP: Begg's test *z* = 3.47, *p* = 0.001, and Egger's test *p* < 0.001; and LVEF: Begg's test *z* = 3.27, *p* = 0.001, and Egger's test *p* = 0.024). We used the trim-and-fill method to recalculate the pooled effect size. A total of 10 and 19 studies, respectively, were added to the funnel plots for 6MWD and LVEF, but the pooled SMD was not affected. For BNP and NT-pro BNP, no new studies were added, but the pooled effect size changed significantly (Figure 5). See Discussion for a more detailed interpretation of these findings. There was no evidence for significant publication bias in analyses of LVEDD and LVESD.

## 4. Discussion

To the best of our knowledge, this is the largest systematic review and meta-analysis of the effect of QSYQ on CHF. Our results indicated that the addition of QSYQ to routine Western medicine might inhibit cardiac hypertrophy and improve cardiac function and exercise tolerance, as evidenced by decreases in LVEDD and LVESD as well as increases in 6MWD, LVEF, and cardiac index.

Qishen Yiqi is a widely used Chinese herbal medicine with a “qi invigorating and blood activating” property [23]. The dripping pill preparation (QSYQ) is a commercial herbal medicine approved by the China Food and Drug Administration (CFDA) in 2003 and is used extensively in clinical settings to treat cardiovascular diseases, such as angina pectoris, and for the secondary prevention of myocardial infarction [24, 25]. Recent studies have explored the effectiveness of OSYQ in patients with CHF [26–28]. A lack of consistency across these studies used to be explained by small sample sizes and differences in treatment courses, among other factors. Only one previous meta-analysis has been published in 2019 by Chang et al. [13]. It included 12 high-quality studies (Jadad ≥ 2) involving 942 patients with CHF and suggested that QSYQ is effective and safe for improving ventricular remodeling and heart function in patients, consistent with the results of our study. Our meta-analysis included 85 studies of 8,579 total patients with CHF, providing much greater statistical power. Besides, we made relatively more sufficient exploration on heterogeneity, such as subgroup analyses according to treatment course and study quality and analyses of heterogeneity based on Galbraith plots, as well as cut-and-fill method.

The current study showed that there were substantial heterogeneity and publication bias among all published literatures in this field. We analyzed all outlier studies identified by the Galbraith plot and found that the high heterogeneity among studies could not be explained by a single factor. It was potentially generated by a combination of factors, including population age, sex, primary diseases, courses of diseases, treatment courses, original cardiac function, and study design. For example, as to application of combined medication, there are 77 studies (77/85) that reported about the routine Western medicine used in their studies, which contains angiotensin-converting enzyme inhibitor (ACEI), loop diuretic (LD), aldosterone receptor antagonists (MRAs), digitalis, and *β*-adrenergic blocker (BB). The remaining eight (8/85) studies did not mention about the detailed definition of routine Western medicine. But as the etiology of heart failure (i.e., ischemic heart disease, valvular heart disease, dilated cardiomyopathy, and hypertensive myocardiopathy, as well as pulmonary heart disease) and complications of patients (i.e., blood pressure disorder and renal dysfunction) enrolled in these studies are different, the actual medicines used in every patient and also across each collected study are not consistent. Moreover, as stated by the included studies, some medicines for specific primary diseases were also used in part of these patients. For instance, patients with ischemic cardiomyopathy also take antiplatelet drugs, statin, and nitrates. This clinical diversity (or clinical heterogeneity) in clinical meta-analysis is inevitable and always unable be explored furtherly, unless more detailed individual information of every patient could be provided by the original studies. In addition, potential publication bias existing in this field and heterogeneity would impact each other when both present, which is not uncommon in many published meta-analysis [29]. Similarly, as indicated, it is unrealistic to reliably distinguish the impact of publication bias and heterogeneity in meta-analysis unless detailed and individualized data are available [30]. For the current study, a total of 85 studies were included, making it impossible to obtain detailed raw data from all these studies. Therefore, we could not make further quantified analysis on the impact of this clinical diversity on the overall heterogeneity. The existence of publication bias and the substantial heterogeneity in the published literatures may temporarily limit the clinical evidence levels and recommendation grades of QSYQ in heart failure at the moment. Besides, the results of 95% prediction interval showed that QSYQ might not always be effective in all clinical cases. Given all the abovementioned, it is suggested that, in future researches, we should focus on the efficacy of QSYQ in a certain type of patients to ensure homogeneity and, at the same time, encourage the reporting of negative results in medication researches. In practical settings, when referring to existing evidences, clinicians should make individualized dialectical therapeutic medication plan according to the specific conditions of patients.

Findings at the cellular and organismal levels tended to support the protective effect of QSYQ in CHF. Wang et al. studied an HF rat model induced by left anterior descending coronary artery ligation and found that QSYQ can exert an antifibrotic effect by downregulating the renin-angiotensin-aldosterone system pathway and subsequently inhibiting the expression of proteins in the arachidonic acid metabolic pathway [31]. Li et al. found that posttreatment with QSYQ obviously suppresses the expression of CD68 and transforming growth factor beta 1, thereby attenuating pressure overload-induced cardiac hypertrophy and myocardial fibrosis [32]. Wang et al. found that QSYQ reduces myocardial fibrosis induced by doxorubicin by promoting cardiac angiogenesis [33]. Zhang et al. showed that 24 combinatorial bioactive ingredients in QSYQ identified through UPLC-Q-TOF/MS significantly prevented myocardial injury; improved the ejection fraction and fractional shortening; decreased the release of cardiac enzymes, including CK, CK-MB, and LDH; alleviated mitochondrial dysfunction; and protected cell nuclei and mitochondrial mass [34]. Potential targets of QSYQ include extracellular signal-regulated kinase-1/2, peroxisome proliferator-activated receptor-gamma and heme oxygenase-1, *β*2-adrenergic receptor, and hypoxia-inducible factor 1*α* (HIF-1*α*) [35–37]. Cui et al. confirmed that QSYQ significantly suppresses myocardial hypertrophy and ventricular remodeling in aortic stenosis-induced HF rats; it is also remarkably better when compared with single herbs [38]. Our previous study of QSYQ also indicated that it has protective effects against apoptosis and inhibits mitochondrial dysfunction [8].

Limitations of this study should be mentioned. First, we did not evaluate the prognostic value of QSYQ in CHF, including effects on mortality and rehospitalization, owing to the lack of available data from primary studies. However, since the parameters included in our study, such as LVEF and cardiac index, are strong predictors of prognosis, our results provide a reference for the prediction of prognosis in CHF [39, 40]. Second, there was high heterogeneity and potential publication bias because the sample sizes of the included studies were generally small. Our results for 6MWD, LVEF, cardiac index, LVEDD, and LVESD were stable in all sensitivity analyses. However, large-scale, multicenter, randomized, double-blind high-quality studies are still needed. Third, all studies included in the meta-analysis were conducted and published in Chinese. This is not surprising, as QSYQ is a traditional Chinese medicine. However, it is necessary to confirm its value in other populations, particularly as traditional Chinese medicines are gradually gaining popularity in Western countries.

## 5. Conclusion

Our current meta-analysis indicated that QSYQ combined with Western therapy might be effective in CHF patients. Further researches are needed to identify which subgroups of CHF patients will benefit most and what kind of combination medicine that works best.

## Figures and Tables

**Table 1:** Summarized results of the meta-analysis in high-quality studies.

Parameters	Category	Studies	Participants	SMD (95% CI)	*p*	*I* ^2^ (%)	Heterogeneity, *p*
6MWD	Overall	11	1065	2.38 (1.63 to 3.13)	<0.001	96	<0.001
	Adjustment by treatment course						
	1-4 wk	1	180	3.94 (3.43 to 4.44)	<0.001	NA	NA
	5-8 wk	5	475	2.13 (1.19 to 3.07)	<0.001	94	<0.001
	<8 wk	5	410	2.31 (1.10 to 3.52)	<0.001	96	<0.001
BNP	Overall	13	1464	-2.90 (-3.76 to -2.03)	<0.001	97	<0.001
	Adjustment by treatment course						
	1-4 wk	2	278	-2.80 (-5.95 to 0.36)	0.08	99	<0.001
	5-8 wk	6	680	-2.70 (-3.77 to -1.63)	<0.001	96	<0.001
	<8 wk	5	506	-3.36 (-5.25 to -1.46)	<0.001	98	<0.001
NT-pro BNP	Overall	6	645	-3.58 (-5.15 to -2.01)	<0.001	98	<0.001
	Adjustment by treatment course						
	1-4 wk	1	178	-0.59 (-0.90 to -0.29)	<0.001	NA	NA
	5-8 wk	2	200	-9.87 (-27.06 to 7.32)	0.26	99	<0.001
	<8 wk	3	267	-1.80 (-3.46 to -0.14)	0.03	97	<0.001
LVEF	Overall	24	2611	1.08 (0.84 to 1.33)	<0.001	88	<0.001
	Adjustment by treatment course						
	1-4 wk	4	610	0.82 (0.19 to 0.45)	0.01	93	<0.001
	5-8 wk	9	962	1.29 (0.86 to 1.71)	<0.001	89	<0.001
	<8 wk	11	1039	1.02 (0.69 to 1.36)	<0.001	84	<0.001
LVEDD	Overall	14	1665	-1.34 (-1.87 to -0.80)	<0.001	96	<0.001
	Adjustment by treatment course						
	1-4 wk	2	330	-1.09 (-3.25 to 1.06)	0.32	99	<0.001
	5-8 wk	7	762	-1.58 (-2.33 to -0.83)	<0.001	95	<0.001
	<8 wk	5	573	-1.10 (-2.04 to -0.15)	0.02	96	<0.001
LVESD	Overall	13	1592	-0.60 (-1.14 to -0.05)	0.03	96	<0.001
	Adjustment by treatment course						
	1-4 wk	2	330	-0.67 (-1.99 to 0.65)	0.32	97	<0.001
	5-8 wk	7	762	-0.28 (-1.12 to 0.55)	0.51	97	<0.001
	<8 wk	4	500	-1.12 (-2.04 to-0.19)	0.02	95	<0.001

6MWD: 6-minute walking distance; BNP: brain natriuretic peptide; NT-pro BNP: N-terminal prohormone of BNP; LVEF: left ventricular ejection fraction; LVEDD: left ventricular end-diastolic dimensions; LVESD: left ventricular end-systolic dimensions.

## Data Availability

All data used in this study have been listed in Supplementary Table S1.

## Supplementary Materials

Supplementary Materials The detailed information of studies included in the current meta-analysis.
