# Supplementary material for: Efficacy of Qishen Yiqi Drop Pill for Chronic Heart Failure: An Updated Meta-Analysis of 85 Studies
Source: Cardiovasc Ther. 2020 Sep 22;2020:8138764. doi: 10.1155/2020/8138764 (PMC7530480; doi:10.1155/2020/8138764)
Supplement: Supplementary Materials — The detailed information of studies included in the current meta-analysis. [file 8138764.f1.docx]

**Table S1 Characteristics of the studies included in the meta-analysis**

| **First author** | **Year** | **Region** | **Numbers**  **Treatment/Control** | **Age (years)**  **Treatment/Control** | **Males**  **Treatment/Control** | **Disease duration (years)**  **Treatment/Control** | **NYHA II/III/IV**  **Treatment group** | **NYHA II/III/IV**  **Control group** | **Treatment course (weeks)** | **Outcomes** | **Jadad score** |
| --- | --- | --- | --- | --- | --- | --- | --- | --- | --- | --- | --- |
| Luo JH | 2007 | Jiangxi | 25/25 | NA | NA | NA | NA | NA | 48 | LVEF | 4 |
| Liao YX | 2008 | Guangxi | 80/40 | 60±7/61±8 | 39/18 | 2.5±10.4/2.4±10.6 | 48/32/0 | 25/15/0 | 4 | LVEF | 2 |
| Tian F | 2008 | Henan | 30/28 | NA | NA | NA | NA | NA | 2 | LVEF/cardiac index | 2 |
| Meng MK | 2010 | Guangxi | 62/64 | 72±11/72±12 | 38/39 | 7.8±1.7/7.7±1.5 | 16/33/13 | 20/31/13 | 8 | LVEF/LVEDD | 2 |
| An YX | 2010 | Henan | 64/64 | 67±4/68±6 | 36/37 | 10+/10+ | 0/51/13 | 0/53/11 | 2 | 6MWD | 2 |
| Lin X | 2010 | Guangxi | 106/106 | 71.3±4.6/70.8±4.3 | 61/62 | NA | 24/53/29 | 25/51/30 | 4 | 6MWD/LVEF/cardiac index | 2 |
| Wang D | 2010 | Liaoning | 89/76 | NA | NA | 0.33-12/0.33-12 | NA | NA | 48 | LVEF | 2 |
| Zhong D | 2010 | Guangxi | 74/74 | 70.5±3.5/69.5±3.0 | 46/48 | NA | 18/32/24 | 17/34/23 | 4 | 6MWD/LVEF/cardiac index | 2 |
| Chen TG | 2011 | Jiangxi | 60/59 | 55.3±10.5/57.7±11.8 | 32/32 | 6.3±2.9/5.8±2.6 | 22/28/10 | 22/28/9 | 12 | BNP/LVEF/LVEDD/LVESD | 2 |
| Chen SR | 2011 | Sichuan | 43/47 | NA | NA | NA | NA | NA | 12 | BNP/LVEF/LVEDD | 2 |
| Cao JY | 2012 | Shandong | 66/66 | NA | NA | NA | 23/39/4 | 25/35/6 | 2 | LVEF | 1 |
| Liu J | 2012 | Henan | 40/40 | 62.3±5.3/61.8±6.4 | 23/24 | NA | 0/29/11 | 0/28/12 | 12 | LVEF | 2 |
| Jia HL | 2012 | Henan | 40/40 | NA | NA | NA | NA | NA | 8 | 6MWD/NT-pro BNP/LVEF | 4 |
| Teng W | 2012 | Henan | 30/31 | 51-89/52-88 | 20/19 | NA | NA | NA | 8 | BNP/LVEF/LVEDD/LVESD | 2 |
| Ren YH | 2012 | Jilin | 62/62 | 30-80/30-81 | 32/30 | 1-6 | 18/26/18 | NA | 4 | LVEF | 2 |
| Wang JX | 2012 | NA | 60/60 | 3.84±0.65 | 26 | 0.416-14 | NA | NA | 4 | BNP/LVEF | 2 |
| Zhang JL | 2012 | Guangdong | 79/79 | 60±5.5/62±2.5 | 40/42 | NA | 0/42/37 | 0/38/41 | 4 | 6MWD/BNP/LVEF | 2 |
| Guan XJ | 2013 | Wuhan | 50/50 | 63.7±9.4/62±10.9 | 30/32 | NA | 12/25/13 | 11/24/15 | 24 | NT-pro BNP/LVEF/LVEDD/LVESD/cardiac index | 2 |
| Qin CH | 2013 | Henan | 60/54 | 69.5±10.6/68.7±9.8 | 37/32 | NA | 14/30/16 | 14/26/14 | 8 | 6MWD/BNP/LVEF | 2 |
| Hua JY | 2013 | Henan | 43/43 | 65/66 | 35/36 | 4+/4+ | 0/0/43 | 0/0/43 | 8 | BNP/LVEF/LVEDD/LVESD | 2 |
| Wu TC | 2013 | Guizhou | 30/30 | 66.12±6.63/65.00±7.96 | 17/16 | 1+/1+ | 22/8/0 | 24/6/0 | 24 | 6MWD/BNP/LVEF | 7 |
| Zhang B | 2013 | Liaoning | 30/30 | 60.34±13.50/60.50±14.48 | 16/20 | 1+/1+ | NA | NA | 12 | 6MWD/BNP/LVEF | 4 |
| Wu B | 2013 | Jiangxi | 60/60 | 59.5/58.6 | 35/36 | 2-15/2-15 | NA | NA | 12 | BNP/LVEF/LVEDD/LVESD | 2 |
| Zhang JL | 2013 | Hebei | 50/50 | 67±6/68±8 | 20/22 | 8.7±5.0/8.6±5.3 | 12/17/21 | 11/19/20 | 24 | 6MWD/BNP/LVEF/LVEDD | 2 |
| Sun DY | 2013 | Heilongjiang | 60/60 | NA | NA | NA | NA | NA | 12 | LVEF/LVESD | 2 |
| Jiao XQ | 2013 | Henan | 62/60 | 67±7.5/65±6.2 | NA | NA | 20/31/11 | 19/29/12 | 8 | 6MWD/BNP/LVEF/LVEDD/LVESD | 2 |
| Zhang ZH | 2013 | Jiangxi | 40/40 | 69.1±8.5/68.7±7.4 | 20/23 | NA | 10/23/7 | 10/23/7 | 12 | BNP/LVEF/cardiac index | 4 |
| Ding SG | 2014 | Anhui | 50/48 | NA | 30/35 | NA | NA | NA | 4 | BNP | 4 |
| Gu MF | 2014 | Jiangsu | 65/65 | 66.8±3.9/67.8±5.6 | 43/38 | NA | 0/53/12 | 0/58/7 | 2 | 6MWD | 2 |
| Yin W | 2014 | Henan | 20/20 | NA | 11/10 | NA | 8/7/5 | 9/7/4 | 24 | 6MWD/BNP/LVEF | 2 |
| Sun YL | 2014 | Henan | 60/60 | NA | NA | NA | NA | NA | 8 | NT-pro BNP/LVEF | 4 |
| Zhao GH | 2014 | Henan | 39/39 | 69.38±10.65/69.71±10.52 | 24/23 | NA | 8/20/11 | 9/18/12 | 4 | 6MWD/BNP/LVEF/cardiac index | 1 |
| Shao B | 2014 | Jiangsu | 36/37 | 68.5±9.6/66.7±11.2 | 22/24 | NA | 8/19/9 | 10/16/11 | 12 | NT-pro BNP/LVEF/LVEDD | 4 |
| Li YF | 2015 | Shandong | 90/90 | NA | NA | NA | NA | NA | 2 | 6MWD | 1 |
| He SL | 2015 | Henan | 40/40 | NA | NA | NA | NA | NA | 24 | BNP/LVEF/LVEDD | 2 |
| Shao ZB | 2015 | Anhui | 35/35 | 62.3±10.9/61.3±9.7 | 20/22 | 2-11/2-11 | NA | NA | 8 | 6MWD/BNP/LVEF/LVEDD/LVESD | 4 |
| Yu CY | 2015 | Zhejiang | 40/40 | 60.5±2.0/60.1±1.1 | 24/23 | NA | 5/20/15 | 6/22/12 | 8 | 6MWD/NT-pro BNP/LVEF/LVEDD/LVESD | 2 |
| Xie F | 2015 | Shanxi | 36/36 | NA | NA | NA | NA | NA | 12 | 6MWD/NT-pro BNP/LVEF/LVEDD/LVESD | 4 |
| Li YZ | 2015 | Hubei | 38/38 | 65±4/64±4 | 21/22 | 2-18/3-17 | 13/16/9 | 12/18/8 | 8 | LVEF | 2 |
| Ma CY | 2015 | Jiangsu | 30/30 | 52.2±5.8/53.8±7.9 | 18/16 | 0.6-12/0.7-15 | NA | NA | 4-8 | BNP/LVEF | 2 |
| Liu SQ | 2016 | Shanxi | 52/50 | 61±10/58±11 | 24/24 | NA | 16/24/12 | 17/22/11 | 24 | 6MWD/NT-pro BNP/LVEF | 1 |
| Wang X | 2016 | Tianjin | 30/30 | 54.52±1.14/53.26±1.27 | 18/16 | NA | NA | NA | 4 | NT-pro BNP | 2 |
| Qiu YH | 2016 | Guangdong | 80/80 | NA | NA | 1-18/1-18 | NA | NA | 12 | 6MWD/BNP | 1 |
| Wang LZ | 2016 | Jiangsu | 20/20 | 55.12±4.45/56.58±4.29 | 9/10 | NA | NA | NA | 12 | 6MWD/BNP | 2 |
| Wang QQ | 2016 | Tianjin | 41/41 | NA | NA | 1-16/1-16 | NA | NA | 2 | 6MWD/LVEF | 1 |
| Wang CR | 2016 | Jiangsu | 50/50 | 66.4±6.4/65.8±6.7 | 24/22 | 2-23 | 25/15/10 | NA | 8 | 6MWD/BNP/LVEF/LVEDD/LVESD | 4 |
| Yuan L | 2016 | Henan | 90/90 | 62±5.8/64±5.2 | 47/42 | NA | NA | NA | 4 | 6MWD/BNP/LVEF | 4 |
| Chen XL | 2017 | Xinjiang | 76/76 | 56.64±7.32/57.75±7.52 | 42/40 | NA | 18/30/28 | 20/31/25 | 4 | LVEF/LVEDD/LVESD | 4 |
| Li P | 2017 | Shanxi | 47/47 | 58.36±5.39/59.14±4.97 | 29/27 | 5.41±0.83/5.53±0.74 | 0/31/16 | 0/32/15 | 8 | BNP/LVEF/LVEDD/LVESD | 4 |
| Hu YC | 2017 | Hubei | 43/42 | 71.46±5.58/71.26±5.41 | 28/25 | 15.41±3.14/15.85±3.22 | 11/21/11 | 10/20/12 | 4 | BNP/LVEF/LVEDD | 1 |
| Ren LF | 2017 | Inner Mongolia Autonomous region | 58/42 | NA | NA | 0.08+/0.08+ | NA | NA | 4 | 6MWD/BNP/LVEF | 2 |
| Yang K | 2017 | Shanxi | 50/50 | 67.88±10.23/68.13±9.98 | 34/37 | NA | 14/24/12 | 12/27/11 | 8 | 6MWD/LVEF/LVEDD/LVESD | 2 |
| Wu P | 2017 | Sichuan | 30/30 | 61.5±2.3/61.8±2.1 | 16/20 | NA | NA | NA | 12 | 6MWD | 2 |
| Xu J | 2017 | Henan | 76/70 | 62.4±11.8/63.8±12.1 | 42/38 | 9.5±4.3 | 0/40/36 | 0/37/33 | 8 | 6MWD/BNP/LVEF | 2 |
| Wei B | 2017 | Liaoning | 30/30 | 66.25±5.63/65.02±5.45 | 15/16 | NA | NA | NA | 8 | LVEF | 2 |
| Tang MX | 2017 | Hunan | 70/70 | 66.04±9.08/65.39±8.99 | 36/38 | 8.11±3.75/8.05±3.67 | 29/41/0 | 27/43/0 | 8 | 6MWD/BNP/LVEF/LVEDD/LVESD | 4 |
| Wei Y | 2017 | Anhui | 95/96 | NA | NA | NA | NA | NA | 8 | BNP/LVEF/LVEDD/LVESD | 4 |
| Zhang XQ | 2017 | Hebei | 48/48 | 61.34±2.84/61.40±2.79 | 26/29 | 2-12/2-13 | 0/29/19 | 0/31/17 | 12 | 6MWD/BNP/LVEF/LVEDD/LVESD | 4 |
| Hua CE | 2017 | Jiangsu | 30/30 | NA | NA | NA | NA | NA | 4 | LVEF/cardiac index | 1 |
| Li RC | 2018 | Guangdong | 105/105 | 62.44±6.73/62.35±6.86 | 63/57 | 2.29±0.41/2.31±0.37 | 34/46/25 | 32/47/26 | 12 | BNP/LVEF/LVEDD/LVESD | 4 |
| Li GL | 2018 | Jilin | 40/40 | 78.4±1.5/77.8±1.2 | 29/27 | NA | NA | NA | 4 | 6MWD/LVEF | 1 |
| Hu YJ | 2018 | Jiangsu | 34/34 | 60.24±3.51/60.29±3.26 | 22/21 | NA | NA | NA | 4 | BNP/LVEF | 2 |
| Wang QD | 2018 | Guangdong | 48/48 | 64.2±6.1/65.3±6.7 | 25/27 | 0.25-5/0.25-7 | NA | NA | 8 | 6MWD/LVEF/LVEDD | 2 |
| Zhang L | 2018 | Henan | 30/30 | 61.1±7.5/61.2±7.6 | 17/18 | 1-15/1-16 | NA | NA | 12 | NT-pro BNP/LVEF/LVEDD/LVESD | 1 |
| Wu BL | 2018 | Shanxi | 50/50 | 65±5.6/64±4.8 | 24/25 | 0.33+ | 10/35/5 | 8/36/6 | 12 | 6MWD/BNP/LVEF/LVEDD | 2 |
| Shen J | 2018 | Jiangsu | 40/40 | 62.34±3.91/61.56±3.68 | 22/24 | NA | NA | NA | 4 | BNP/LVEF/LVEDD | 1 |
| Meng ZL | 2018 | Henan | 37/37 | 69.81±7.64/70.03±7.51 | 21/22 | NA | 0/25/12 | 0/24/13 | 8 | NT-pro BNP/LVEF/LVESD | 1 |
| Zeng J | 2018 | Shanxi | 35/35 | 78.54±6.25/78.56±6.23 | 19/20 | 0.83-3/0.67-2 | NA | NA | 12 | LVEF | 4 |
| Chen M | 2018 | Shanghai | 61/61 | 69.9±7.9/69.8±8.3 | 33/32 | 86.89 | NA/NA/6 | NA/NA/6 | 12 | 6MWD/NT-pro BNP/LVEF/LVEDD/LVESD | 4 |
| Che QF | 2018 | Heilongjiang | 50/50 | 61.43±10.66/64.12±11.34 | 27/29 | 0.08-5/0.25-6 | 15/16/19 | 13/17/20 | 4 | LVEF | 4 |
| Mao BY | 2018 | Henan | 60/60 | 68.2±9.7/68.1±9.4 | 35/34 | 3-15/3-15 | 16/31/13 | 17/30/13 | 4 | NT-pro BNP/LVEF/LVEDD/LVESD | 2 |
| Ma TF | 2019 | Henan | 73/73 | 55.69±4.87/54.51±4.36 | 36/38 | 5.71±1.02/5.68±1.05 | 39/34/0 | 40/33/0 | 12 | LVEF | 4 |
| Hu QS | 2019 | Henan | 41/41 | 60.34±7.21/59.89±6.02 | 26/29 | NA | NA | NA | 8 | LVEF/LVEDD/LVESD | 4 |
| Wang JW | 2019 | Jiangsu | 27/26 | 66.24±5.57/65±5.41 | 15/14 | NA | NA | NA | 8 | NT-pro BNP/LVEF | 2 |
| Zou J | 2019 | Anhui | 38/38 | 61.38±5.71/61.52±5.81 | 21/20 | 1-6/1-6 | 7/9/13 | 8/11/11 | 4 | BNP/LVEF/LVEDD/LVESD | 2 |
| Zhang BZ | 2019 | Guangdong | 40/40 | 65.36±7.33 | NA | 10.32±3.25 | NA | NA | 12 | 6MWD/BNP/LVEF | 2 |
| Wang W | 2019 | Shandong | 40/40 | 69.5±9.8/68.8±9.6 | 24/23 | NA | NA | NA | 12 | LVEF | 2 |
| Xu L | 2019 | Jiangsu | 15/15 | NA | NA | NA | NA | NA | 8 | 6MWD/BNP/LVEF | 1 |
| Liu YG | 2019 | Hebei | 27/27 | 64.77±6.49/63.53±7.63 | 15/14 | 3-14/3-15 | NA | NA | 4 | LVEF/LVEDD | 2 |
| Niu HM | 2019 | Shanxi | 53/53 | 57.19±6.01/56.91±5.78 | 26/29 | 1.4-5.9/1.6-6.1 | 22/15/16 | 24/16/13 | 12 | LVEF/cardiac index | 2 |
| Liu NR | 2019 | Henan | 89/89 | NA | NA | NA | NA | NA | 1 | NT-pro BNP/LVEF/LVEDD/LVESD | 4 |
| Jin H | 2019 | Jilin | 45/40 | 57.46±5.15/58.72±5.22 | 25/25 | NA | NA | NA | 8 | 6MWD/BNP/LVEF/LVEDD/LVESD | 4 |
| Liu T | 2019 | Hebei | 54/54 | NA | NA | NA | NA | NA | 4 | 6MWD/NT-pro BNP/LVEF/LVEDD | 1 |
| Lv XB | 2019 | Shanxi | 49/49 | NA | NA | NA | NA | NA | 48 | 6MWD/NT-pro BNP/LVEF/LVEDD | 2 |
| Li XH | 2020 | Jiangxi | 62/63 | 72±10.4/72±9.6 | 38/34 | 7.8±1.7/8.0±1.5 | 9/42/11 | NA | 24 | 6MWD/LVEF/LVEDD/LVESD | 2 |

Notes: 6MWTD, 6-minute walking distance; BNP, brain natriuretic peptide; NT-pro BNP, N-terminal prohormone of BNP; LVEF, left ventricular ejection fraction; LVEDD, left ventricular end-diastolic dimensions; LVESD, left ventricular end-systolic dimensions.
